# Progressive Vision Loss in a Patient With Axenfeld-Rieger Syndrome

**DOI:** 10.7759/cureus.25128

**Published:** 2022-05-18

**Authors:** Taimoor A Khan, Muhammad A Zahid, Amjad Akram, Abdul Rauf

**Affiliations:** 1 Ophthalmology, Armed Forces Institute of Ophthalmology, Rawalpindi, PAK; 2 Emergency Medicine, Casey Hospital, Monash Health, Berwick, AUS

**Keywords:** axenfeld-rieger syndrome, genetic counseling, autosomal dominant genetic disorder, glaucoma, vernal keratoconjunctivitis

## Abstract

Axenfeld-Rieger syndrome (ARS) is a rare autosomal dominant condition characterized by the dysgenesis of the anterior segment along with some systemic abnormalities such as dental and facial bone defects. Its incidence is thought to be 1 in 200,000. Treatment is predominantly the management of glaucoma and is mostly medical but can be surgical in refractory cases. Here, we describe the case of a 35-year-old female patient who presented with co-existing vernal keratoconjunctivitis and ARS. The treatment was more challenging as we had to manage two conditions simultaneously.

## Introduction

Axenfeld-Rieger syndrome (ARS) is a rare autosomal dominant condition characterized by the dysgenesis of the anterior segment along with some systemic abnormalities. Axenfeld anomaly was first described by the German Ophthalmologist Theodore Axenfeld in 1920 as the eye having peripheral anterior segment defects characterized by posterior embryotoxon and iris strands adherent to the anteriorly displaced Schwalbe’s line [1]. In 1934, Rieger described changes in the iris, namely, polycoria, corectopia, and hypoplasia, which today are termed Rieger anomaly [2]. Rieger anomaly combined with systemic features such as dental and facial bone defects came to be known as Rieger syndrome. All of these signs and symptoms when present in a single patient are termed as ARS. Its incidence is thought to be 1 in 200,000 [3], but it may be higher in countries where consanguineous marriages are more common. It can present with a variety of clinical features. Glaucoma is present in approximately 50% of cases. Treatment is predominantly the management of glaucoma and is mostly medical but can be surgical in refractory cases. Here, we describe the case of a 35-year-old female patient who presented with co-existing vernal keratoconjunctivitis (VKC) and ARS. The treatment was more challenging as we had to manage two conditions simultaneously.

## Case presentation

**Figure 1:**
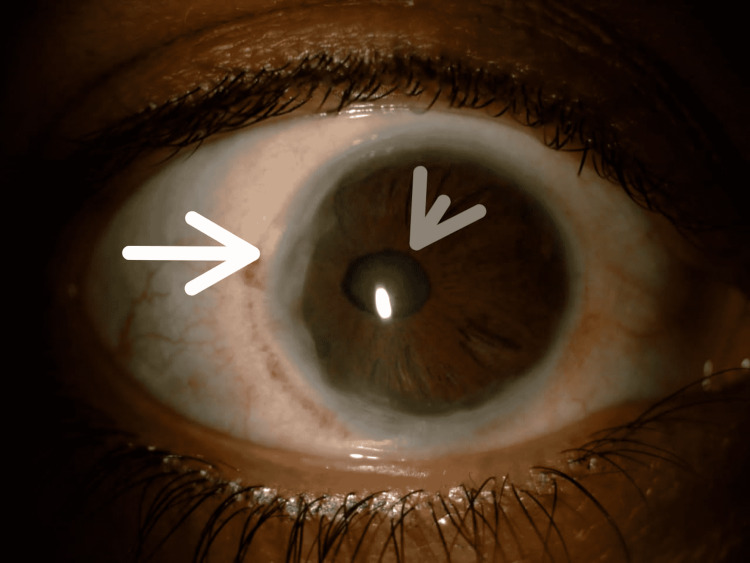
Anterior segment photograph of the right eye showing posterior embryotoxon (peripheral anterior synechiae, white arrow), corectopia, and an oblong pupil with adjacent iris atrophy (gray arrow).

**Figure 2:**
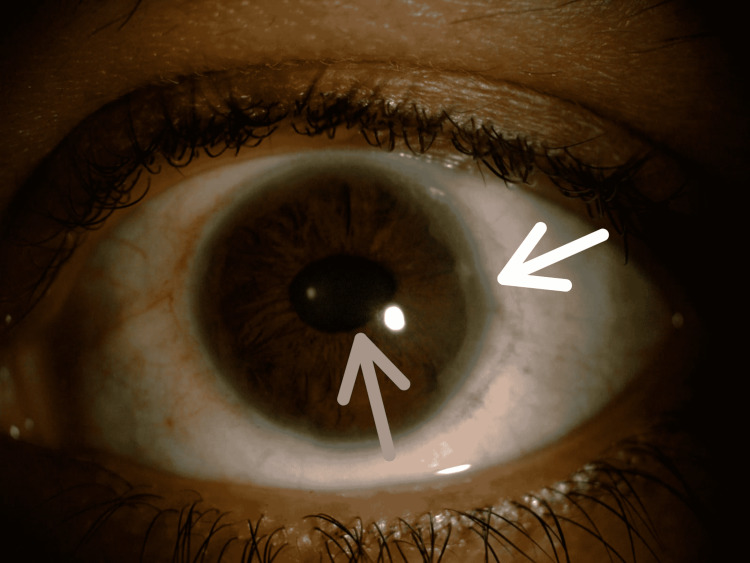
Anterior segment photograph of the left eye showing posterior embryotoxon (peripheral anterior synechiae, white arrow), corectopia, and oblong pupil with adjacent iris atrophy (gray arrow).

**Figure 3:**
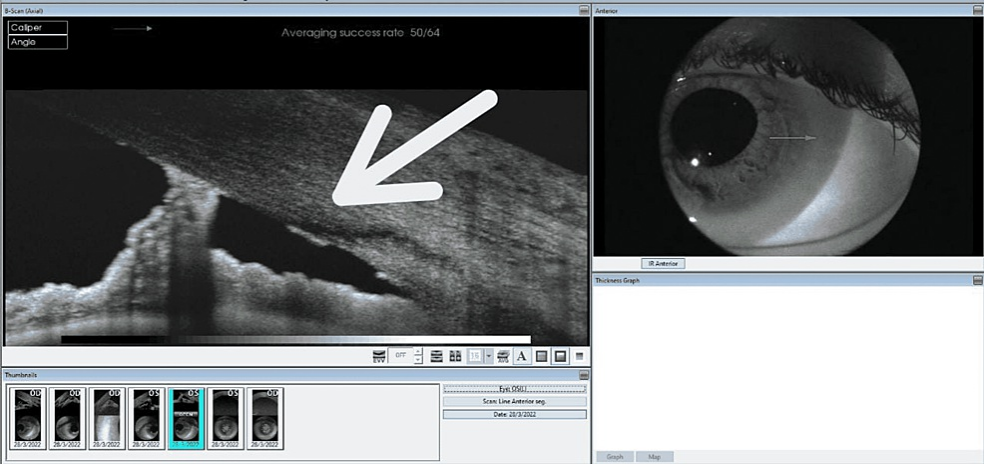
Anterior segment optical coherence tomography of the left eye showing iridocorneal touch but an open anterior chamber angle (white arrow).

**Figure 4:**
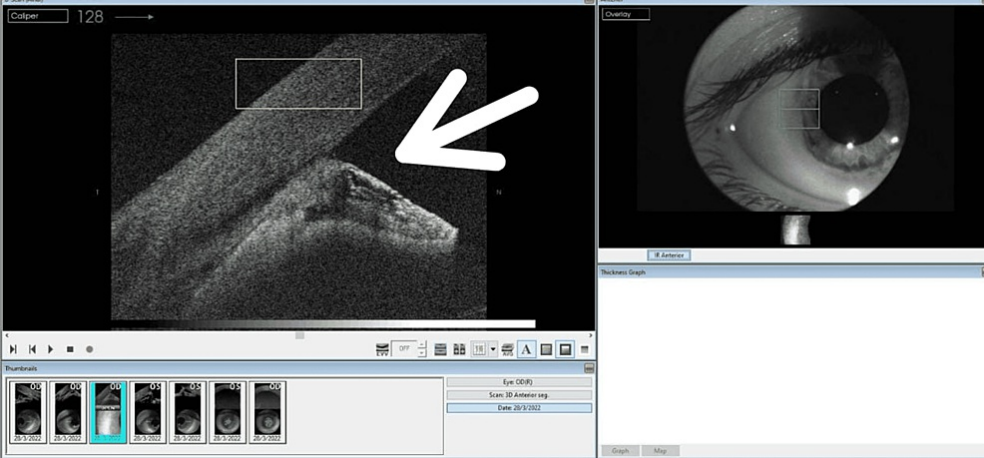
Anterior segment optical coherence tomography of the right eye showing shallow anterior chamber with iridocorneal contact and completely occluded anterior chamber angle (white arrow).

A 35-year-old mother of two children presented with complaints of gradually progressive painless loss of vision in her right eye. She had been using glasses since the age of seven, and her number had recently changed. Her unaided visual acuity in the right eye was 6/12, best-corrected to 6/12 with (-5.50 × 90 degrees) refraction. Her unaided visual acuity in the left eye was 6/12, best-corrected to 6/6 with (-2.00 × 93 degrees) refraction. Her examination revealed bilateral maxillary hypoplasia, malar rash, and crowding of all teeth (both maxillary and mandibular). Adnexal examination revealed bilateral papillae at the upper tarsal conjunctiva. Anterior segment examination showed bilateral bluish-tinged sclerae, prominent corneal nerves, posterior embryotoxon from 8-11 o’clock and 2-5 o’clock in the right eye and 9-11 o’clock and 1-6 o’clock in the left eye. Iris showed bilateral atrophic patches, and pupillary examination revealed oval-shaped nasally displaced pupils (corectopia); however, they were regular and equally reactive to direct light. There was no Marcus Gunn pupil. Pupils had poor mydriasis in response to dilation with topical 0.1% mydriatic eye drops. Posterior segment examination was unremarkable in both eyes. Her intraocular pressure (IOP) by Goldman applanation tonometer (GAT) was 14 mmHg in the right and 16 mmHg in the left eye. Her gonioscopy revealed bilateral posterior embryotoxon with Shaffer’s grade IV open angles. Videokeratography (VKG) of both eyes revealed findings suggestive of early keratoconus in both eyes and a central corneal thickness of 540 µm in the right and 546 µm in the left eye. Her Humphrey’s visual field 30-2 (SITA fast protocol) using Humphrey’s Field Analyzer 3 (v 1.5.2.431) did not reveal any scotoma/field defect. We kept our patient on follow-up and recommended a classical water drinking test on her next visit. After taking a baseline reading of IOP before the patient drank 1 L of water, we examined the patient four times for IOP measurement by GAT every 30 minutes. The baseline was 15 and 17 mmHg, and at two hours, the patient had IOP of 20 and 21 mmHg. Based on clinical examination and supporting ocular investigations, her diagnosis of VKC with Axenfled-Rieger anomaly was established. A trial of glasses was advised along with a combination of topical antihistamines and mast cell stabilizers (twice daily), preservative-free topical lubricants (four times daily), and oral anti-histamines when needed. After we ruled out the chances of raised IOP in our patient, we advised a six-monthly follow-up for a detailed ocular examination. Corneal cross-linkage was also kept in the plan if any further progression was evident on the next visit (Figures 1-4).

## Discussion

ARS is a rare autosomal dominant condition in which posterior embryotoxon and iris strands adhere to the anteriorly displaced Schwalbe’s line. Several genes are thought to play a role in its pathogenesis, namely, *PITX2*, *FOXC1*, *PAX6*, and *FOXO1A* [3]. These genes, in turn, are responsible for different clinical features in patients and have recently led this disease to be classified into ARS types 1, 2, and 3 [4-6]. These range from anterior chamber and iris defects more commonly to glaucoma, hearing impairment, and abnormal cardiovascular system morphology, which may be present in 50% of people. Less common features include maxillary hypoplasia and blindness. While anterior segment defects and iris abnormalities were present in our patient, less common features such as maxillary hypoplasia and progressive visual loss were also seen in our patient. Malar rash was also seen which has not been reported before as being a part of ARS. Our patient had no history of an autoimmune condition. Furthermore, bilateral papillae were also seen in the upper tarsal conjunctiva. These combined with her symptoms of severe itching, mucous discharge, and blurring of vision led us to suspect the co-existence of VKC. This was confirmed on anterior segment optical coherence tomography and videokeratography. We also believe that the co-existence of VKC with ARS has only been reported once in the medical literature [7]. Persistent rubbing of the eyes in VKC patients can lead to the development of keratoconus [8], which we also observed in our patient. Hence, we intend to raise awareness about the simultaneous existence of a vision-threatening ocular pathology (VKC) with ARS which would make the management of a patient even more challenging.

Because 50% of patients with ARS present with glaucoma, the management of ARS typically involves managing glaucoma. Glaucoma is usually managed with beta-blockers, carbonic anhydrase inhibitors, and prostaglandin analogs. Recently, alpha-2 agonists have also been used [3]. Surgical management is thought to be more efficacious than medical management and includes goniotomy, trabeculotomy, and trabeculectomy. Although glaucoma drainage devices offer similar efficacy as trabeculectomy, the incidence of endophthalmitis as a complication is far lower [3]. Because IOP was within the normal range in our patient, no treatment for glaucoma was initiated. However, the simultaneous presentation of VKC and early findings of keratoconus made it imperative to start the treatment for VKC. Some of the most popular treatments are thought to be conservative management (cool compresses and lid scrubs and topical antihistamines in mild cases) [9]. Topical antihistamines are also sometimes combined with topical mast cell stabilizers for moderate VKC [9]. We used all four of these treatments in addition to oral antihistamines and noted a favorable response in our patient on the three-month follow-up. It is thought that oral antihistamines may not play a significant role in the treatment of VKC [8]; however, this was contrary to what we experienced. It is also thought that oral and injectible corticosteroids [9], and several immunomodulators such as topical cyclosporin and tacrolimus [10,11] may be needed for long-term management. While this may be true, we did not have a chance to observe their effects as our patient responded satisfactorily to the combination of topical antihistamines and mast cell stabilizers in addition to oral antihistamines. However, the effect of immunomodulators may be observed in our patient if she shows disease progression at the six-month follow-up. Furthermore, we believe that genetic counseling is often not provided to patients with ARS. Being an autosomal dominant condition, it is imperative that people in countries where consanguineous marriages are more common are provided familial and genetic counseling. We advocate that this be a compulsory part of the management of ARS. We also advocate that a more stringent and shorter follow-up time (three months) be kept in patients who present with a simultaneous vision-threatening ocular pathology (VKC) along with ARS.

The limitation of this case report is its inherent nature of being a single case. Furthermore, due to resource constraints, no genetic evaluation of this patient and her family members was done, which would have given more insight into the possible pathogenesis of this disease.

## Conclusions

ARS is a rare autosomal dominant condition that presents with anterior segment dysgenesis and several systemic abnormalities. We believe that the presence of VKC with ARS, a presentation reported in literature only once before, makes the treatment of ARS more challenging. As such, we advocate a shorter follow-up time than usual. Furthermore, it is very important to stress the role of familial and genetic counseling in the management of ARS, especially in countries where consanguineous marriages are more common.
